# Structural characteristics of carbon information disclosure networks and the impact on carbon emission performance: Evidence from China

**DOI:** 10.1016/j.heliyon.2024.e36938

**Published:** 2024-08-29

**Authors:** Tianjiao Jiang, Hua Li, Qiubai Sun

**Affiliations:** School of Business Administration, University of Science and Technology Liaoning, Anshan, 114051, China

**Keywords:** Carbon information disclosure, Carbon emission efficiency, Social network analysis, Undesirable slacks-based measure model

## Abstract

Improving corporate carbon emission performance is an important driving force for realising the low-carbon development of China's economy. Carbon information disclosure is an environmental regulatory tool proposed in the context of carbon neutrality, this study examines whether carbon information disclosure effectively influences carbon emission performance. Based on the data from 30 provinces in China from 2012 to 2021, this paper examines the structural characteristics of carbon information disclosure using social network analysis, accounting for the carbon emission performance of industrial enterprises in each province in China. Using the spatial econometric model, we apply the undesirable slacks-based measure model and examine the impact of the spatial network characteristics of carbon information disclosure on carbon emission performance. Results show that the overall density of carbon information disclosure networks of industrial enterprises in Chinese provinces is low, so there is much room for improvement. Additionally, there is spatial dependence on carbon emission performance among neighbouring provinces. Moreover, the out-degree centrality and betweenness centrality of carbon information disclosure networks significantly negatively affect carbon emission performance. However, the inhibitory effect of the in-degree centrality of carbon information disclosure networks on carbon emission performance is not significant. The unique findings of this paper are relevant to environmental policy formulation and assessment.

## 1 Introduction

Global climate change and sustainable development are critical global challenges. As the world's largest CO_2_ emitter, China plays a vital role in global climate governance and reducing energy consumption and environmental pollution has become an urgent issue.

Carbon information disclosure connects enterprises and the public in the interaction of climate change, which is conducive to the coordinated development of economic growth, energy saving and emission reduction. Under the carbon peaking and neutrality goals, establishing of a standardised mandatory system for corporate carbon information disclosure has become an inevitable trend; however, China's carbon information disclosure is still in the early stage of development, and its carbon information disclosure policy suffers from a low self-disclosure rate and uneven levels of disclosure quality. Simultaneously, improving carbon emission performance is an important criterion for evaluating and reflecting the effectiveness of the government's energy-saving and carbon-reduction policies. Industry is an important source of carbon emissions, and industrial enterprises are the main contributors to increased carbon emissions. Effectively solving the carbon emission problems of industrial enterprises is of great significance for improving the country's overall carbon emission performance. Analysing the current situation of carbon information disclosure in China and the policy effects of carbon information disclosure on improving carbon emissions requires further study of the relationship between carbon information disclosure and carbon emission performance. Such investigation can provide policy recommendations for environmental protection and sustainable development.

Carbon information disclosure usually refers to the public disclosure of information on greenhouse gas emissions (represented by carbon dioxide) generated by enterprises in their production and operation activities. This information is shared with investors and the public through periodical reports or interim reports. According to the different mandatory degrees, carbon information disclosure is divided into mandatory and voluntary carbon information disclosure. How information is reported to stakeholders has important implications for programme effectiveness [1]. Mandatory carbon disclosure has the advantage of comparability, and its quality is better than voluntary carbon information disclosure; however, investors are more concerned about the content of voluntary carbon information disclosure because it helps them more in their investment decisions [2]. Existing literature on carbon information disclosure focuses on its corporate influencing factors and financial consequences. Regarding influencing factors, some literature focuses on internal factors such as independent directors [3] and the percentage of female personnel (female executives and female employees) [4]. In contrast, some studies find that external factors such as social and financial markets [5], external investors [6] and legitimacy pressure [7] can affect carbon information disclosure. Studies on the financial consequences of carbon information disclosure have mainly focused on its impact on firm value. For example, Wang et al. found that environmental information disclosure had positively affected financial performance in a study of 289 Chinese listed companies [8]. Zhang et al. examined the impact of environmental information disclosure on economic performance in China at the firm level, determining that the impact is not the same in the short and long term (negative in the short term, positive in the long term) [9]. Most extant studies default to the independence of carbon information disclosure among research subjects; few studies [10] consider the spatial dissemination and interactive effects of carbon emission information. Spatial interactions in carbon information dissemination exist due to the clustering and association of economic factors. Moreover, spatial effects of environmental policies arise [11], and decentralised environmental policies can lead to a ‘race to the bottom’ [12] and a ‘race to the top’ [13], and strategic interactions occur in policy formulation and implementation among local governments. This situation requires that the interaction between carbon information disclosures among different provinces be considered when analysing the impact of carbon information disclosures on environmental performance. The relative positions of different regions in the overall network of carbon information disclosure and their sending and receiving of carbon information should be examined. Furthermore, a systematic investigation of the spatial correlation effect of regional carbon information disclosure in China should be conducted. These approaches are conducive to understanding the characteristics of China's spatial network of carbon information disclosure. They can provide theoretical support for the government to improve the policy of carbon information disclosure from the whole to the local level.

Existing studies mainly use single-factor indicators [14,15] and total-factor indicators [[16], [17], [18]] to account for carbon emission performance. Single-factor carbon emission performance measures the carbon dioxide emissions per unit of economic output; the indicators used are mainly carbon emission intensity [19] and carbon productivity [20]. The total-factor carbon emission performance mainly measures the relative relationship between multiple factor inputs and outputs and desired outputs, and the commonly used methods to measure carbon emission performance include data envelopment analysis (DEA) and stochastic frontier approach (SFA). SFA needs to set a specific production function and can only study a single output. In comparison, DEA does not need to set a specific form of function; however, it can avoid the structural bias caused by the mis-setting of the production function of SFA. SFA is suitable for multi-input and multi-output performance evaluation; thus, scholars mostly use DEA for carbon emission performance evaluation. The research perspective on carbon emission performance mainly focuses on the regional level. For example, Wang et al. explored the spatial and temporal dynamics of China's carbon emission performance at the city level [21]. Shao et al. measured the carbon emission performance of 30 provinces, investigating the impacts of economic restructuring and green technology progress on China's low-carbon transition development [22]. Shao et al. examined the impact of zombie enterprises in Chinese cities at the prefecture level and above on the development of low-carbon transition in Chinese cities from the perspective of carbon emission performance [23]. Pan et al. examined the effect and impact mechanism of Internet development on urban carbon emissions at the urban level [24]. Zhou and Li used panel data from 271 Chinese cities to study the effects of two policy combinations, new energy demonstration cities and low carbon cities, on carbon emission performance [25]. Established studies usually take the overall region as the research object, which cannot effectively reflect the unique characteristics of a specific industry. The non-expected output SBM model is mainly used to measure the carbon emission performance of industrial industries in a specific region to avoid the one-sidedness of single-factor indicators.

Few studies directly investigate the relationship between carbon information disclosure and carbon emission performance, and most research focuses on the relationship between environmental disclosure and firm value [[26], [27], [28], [29], [30]], the relationship between environmental regulation and economic development [[31], [32], [33]] and the relationship between ecological interventions and green development [34,35]. Some literature suggests that environmental policies can bring environmental benefits and positively influence environmental governance. For example, scholars have used Chinese industrial enterprises as research subjects from the perspective of energy economics and found that promoting eco-friendly technologies and innovation capabilities can effectively improve environmental quality [34]. Moreover, technology and renewable energy positively affect energy efficiency and environmental sustainability [35]; however, the literature has also argued that an ‘inverted U’ relationship exists between environmental regulation and productivity [36]. Therefore, the role of environmental policies on economic development and environmental governance requires further exploration. Moreover, few studies directly investigated the relationship between carbon information disclosure and emission performance. Social network theory states that the direct or indirect relationships between actors form a complex network structure and that the nodes in the network are essential in their access to and transmission of information. Nodes occupying a centrality position have more access to information and can get a head start in information acquisition [37]. Provinces in a better position in the carbon disclosure network have more access to favourable information and occupy an advantageous position, which has a greater impact on carbon emission performance. Therefore, if carbon information disclosure can positively affect carbon emission performance in information transfer, then the nodes occupying a dominant position are transmitting this effect. Conversely, if this effect is negative, the overall network is transmitting this negative effect. Furthermore, according to ‘Tobler's First Law’, the economies of provinces are widely connected, and the economic characteristics of neighbouring regions have strong spatial correlation; however, most studies overlooked the important influence of spatiotemporal factors when exploring the impact of environmental policies on economic development [27,28,33].

Overall, several research gaps remain in the extant literature. First, the relevance of carbon information disclosure among different research subjects is ignored. Different subjects play different roles in the process of carbon information disclosure. Few studies have analysed the network characteristics of carbon information disclosure when conducting related research on carbon information disclosure. Second, existing studies usually consider the region as the object of study, which cannot effectively reflect the unique characteristics of a specific industry. Industrial enterprises have unique characteristics, and improving industrial carbon emission performance may play a crucial role in improving the country's overall carbon emission performance. Third, the spatial spillover effect of carbon emission performance is ignored, i.e. whether the effect of carbon information disclosure spatial correlation network characteristics on carbon emission performance considers the spillover effect of carbon emission performance.

Given the above gaps, this paper systematically characterises the structural features of the spatial correlation network of carbon information disclosure. We examine the impact of the structural features of the spatial correlation network of carbon information disclosure on carbon emission performance. The main objectives of this study are as follows. First, we analyse carbon information disclosure in specific provinces in the context of a national network of spatial correlations of carbon information disclosure. This study aims to understand the relative position of provinces in the carbon information disclosure network and the overall characteristics of the network, which plays a vital role in the country's realisation of clarifying the responsibilities of different regions and the significant contributors to environmental pollution management. Second, this study examines the spillover effects of spatially linked network characteristics of carbon information disclosure on carbon emission performance, aiming to understand the interaction of spatial factors on carbon information disclosure networks and their effects on carbon emission performance. This approach has important implications for strengthening inter-regional factor linkages and realising regional cooperation in managing environmental pollution.

This paper has two advantages over the existing literature. In terms of research content, first, this study constructs a directed carbon information disclosure network to examine the influence of spatial factors on forming a carbon information disclosure network, filling the gap in existing research. Second, considering the influence of spatial spillover effects, this study examines the impact of carbon information disclosure on carbon emission performance. This approach helps the Chinese government understand the effect of environmental policies on high-quality economic development. Regarding research methodology, on the one hand, the social network analysis method is used to examine the spatial network characteristics of carbon information disclosure. This approach allows us to analyse the spatial interaction situation and dynamic evolution characteristics of carbon information disclosure more closely to reality. On the other hand, the use of spatial econometric model to test the relationship between carbon information disclosure and carbon emission performance can better understand the influence of spatial factors on the relationship between the two, and help to understand the competition and cooperation between local governments in the process of environmental governance and economic development.

The remainder of this paper is organised as follows. Section 2 presents theoretical analyses and research hypotheses. Section 3 demonstrates the research methodology. Section 4 explains the empirical results. Section 5 further discusses of the findings; Section 6 gives conclusions and recommendations.

## 2 Theoretical analysis and research hypothesis

Corporate carbon information disclosure is an important manifestation of corporate social responsibility. Corporate social responsibility mainly refers to enterprises' willingness, behaviour and performance to manage their operations' impact on society, stakeholders and the natural environment and to maximize social welfare during their expected lifespan [38]. On the one hand, based on the signalling theory, corporate carbon information disclosure is conducive to alleviating the information asymmetry between enterprises and stakeholders [39], promoting positive interaction between enterprises and stakeholders, and increasing enterprise value. Enterprises can only promote improving overall social value by actively undertaking social responsibility for carbon information disclosure. On the other hand, the theory of shareholder value maximisation holds that the maximisation of shareholder value is the criterion for business decision-making. Furthermore, over-investment of resources in social responsibility by enterprises will result in the loss of shareholders' wealth and enterprise value [40], which is not conducive to improving enterprises’ carbon emission performance. Moreover, the improvement of carbon emission performance is characterised by high up-front investment costs and slow results of environmental protection policies, which will make enterprises face the risk of high environmental expenditures without attractive returns.

Carbon information disclosure, as one of the means of environmental regulation, has both regressive and reverse effects on improving carbon emission performance. On the one hand, new classical economics advocates the ‘follow the cost’ effect, which holds that carbon information disclosure will increase enterprises' costs. Furthermore, the pollution control cost and green technology investment accompanying carbon information disclosure will crowd out the investment in innovation and research and development (R&D) of non-environmental protection sectors, reducing the competitiveness of enterprises; thus, the effect of carbon information disclosure on the performance of carbon emissions will be significantly reduced [41], that is, the ‘regressive’ effect. On the other hand, under the theoretical framework of ‘Porter's hypothesis' of the ‘reverse’ effect [42], a carbon information disclosure policy can help to push enterprises to carry out green technological innovation and improve the efficiency of resource utilisation, and the ‘innovation compensation’ effect can offset and even exceed the ‘follow the cost’ effect, which makes up for the environmental cost brought by carbon information disclosure. Furthermore, the carbon information disclosure policy raises industry entry barriers for enterprises and helps eliminate traditional high-pollution and high-energy-consumption enterprises, thus promoting the improvement of the carbon emission performance of the whole industry.

From the perspective of spatial effects, according to ‘Tobler's first law’ [43], the data of neighbouring regions have specific spatial correlation, and the carbon emission performance of neighboriung regions has spatial spillover effects. Under the influence of local government competition, environmental regulation in the region and neighbouring regions will significantly promote carbon emissions, triggering the ‘race to the bottom’ effect and the ‘green paradox’ of environmental regulation competition [44]. Polluting industries are clustered and tend to agglomerate in areas with weak environmental regulations. According to the ‘pollution heaven hypothesis’ [45], compared with less developed regions, developed regions will set strict and perfect environmental protection standards; hence, the polluting industries in developed regions to avoid such adverse effects will transfer polluting industries to less developed regions with low environmental costs and lax environmental regulations. Environmental regulation triggers a nearby pollution diversion, mainly shown in cities within a 150 km radius [46]. Therefore, the variability of carbon information disclosure in different regions leads to the transfer in and out of polluting industries. This situation affects the improvement of carbon emission performance and makes the spatial dependence of carbon emission performance in neighbouring regions. Based on the above analyses, this paper proposes the following hypotheses.

H1 There is a spatial correlation between the carbon emission performance of neighbouring regions.

H2 When enterprises actively commence social responsibility or the ‘reverse’ effect is dominant, corporate carbon information disclosure network positively impacts corporate carbon emission performance.

H3 When enterprises negatively commence social responsibility or the ‘regressive’ effect is dominant, the corporate carbon information disclosure network negatively impacts corporate carbon emission performance.

**Fig. 1:**
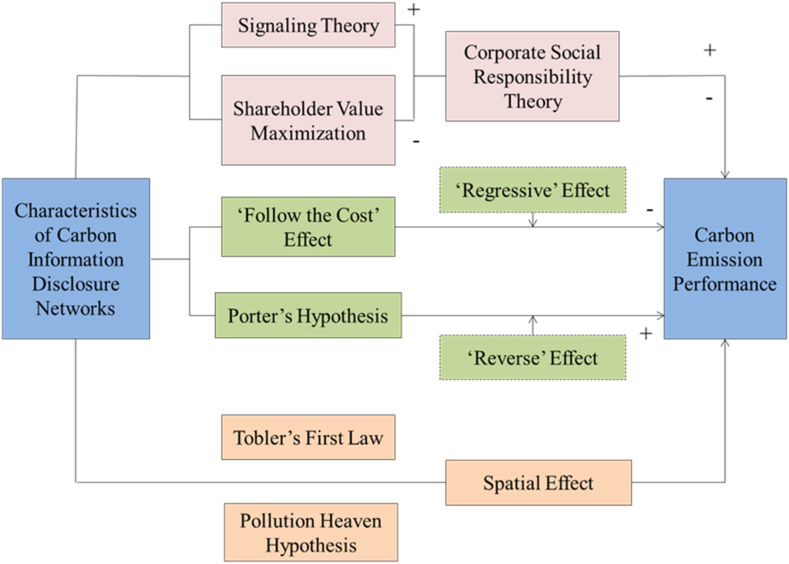
Theoretical mechanism.

The specific theoretical mechanism is shown in Fig. 1.

## 3 Research design

### 3.1 Model specification

#### 3.1.1 Carbon information disclosure spatial association network

This paper selects 30 provincial regions in mainland China (Tibet is excluded due to the large amount of missing data) as the research samples. Each provincial region is used as a node in the network. Furthermore, the spatial network of carbon information disclosure of Chinese industrial enterprises is constructed based on the 30 node regions. Drawing on existing studies [47,48], this paper adopt a modified gravity model to characterise the spatial correlation network between carbon information disclosure in different regions. The specific calculation is shown in the following equations:

(1) $$x_{ij}=k_{ij}\frac{\sqrt[{3}]{P_{i}E_{i}G_{i}}\sqrt[{3}]{P_{j}E_{j}G_{j}}}{D_{ij}^{2}}$$

(2) $$k_{ij}=\frac{E_{i}}{E_{i}+E_{j}}$$

(3) $$D_{ij}=\frac{{d_{i}}_{j}}{g_{i}-g_{j}}$$

where *x*_*ij*_ denotes the spatial correlation strength of carbon information disclosure between regions *i* and *j*. *P*_*i*_ and *P*_*j*_ respectively denote the year-end population of regions *i* and *j*. *G*_*i*_ and *G*_*j*_ respectively denote the real gross domestic product (GDP) of regions *i* and *j*, while *E*_*i*_ and *E*_*j*_ respectively denote the carbon information disclosure (CID), characterised by the carbon information disclosure score. The CID at the provincial level is reflected by the average of the CID scores of A-share industrial enterprises listed in Shanghai and Shenzhen stock in the province. *k*_*ij*_ denotes the contribution rate of region *i* to the strength of the association of CID between regions *i* and *j*. *D*_*ij*_ is the economic geographic distance between regions *i* and *j*. *d*_*ij*_ denotes the geographic distance between regions *i* and *j*. The geographic distance between the two provinces is expressed by the distance between the capital cities of the two regions. Finally, *g*_*i,*_ and *g*_*j*_ respectively denote the per capita GDP of regions *i* and *j*.

The spatial correlation matrix of carbon information disclosure can be obtained from the above gravity model, and each element in the matrix reflects the strength of the correlation of carbon information disclosure between the two corresponding regions. This paper compares the values of the elements in each row of the matrix with the average value of the elements in which they are located and the value higher than (or equal to) the average value of the row, which is recorded as 1. This value means that carbon information disclosure in the region corresponding to that row significantly affects that in the region corresponding to that column. A value lower than the average value of the row is recorded as 0, which means that the carbon information disclosure of the corresponding region in the row does not significantly affect that of the corresponding region in the column. Therefore, the oriented 0–1 network matrix is obtained.

This paper takes 30 provincial regions as nodes, and the association formed by two provincial regions is taken as the connecting line between the nodes. The spatial association strength of carbon information disclosure obtained by the gravity model is taken as the basis to form a 0–1 spatial association network. UCINET software is utilised for overall network analysis and individual network analysis. This paper mainly uses network density for overall network analysis; degree centrality and betweenness centrality are used for individual network analysis. Since closeness centrality has higher requirements for network completeness and is less frequently used, degree centrality and betweenness centrality are mainly calculated.

#### 3.1.2 Spatial econometric models

This paper uses the spatial econometric model to explore the impact of the spatial network characteristics of carbon information disclosure on carbon emission performance. The classical spatial econometric models are mainly categorised into the following three forms.

Spatial autoregressive model (SAR): The SAR model considers that the dependent variables of neighbouring regions may be interdependent. This study shows that changes in local carbon emission performance are affected by neighbouring regions’ carbon emission performance. The SAR model is expressed as

(4) $$Y_{\text{it}}=\rho \sum_{j=1}^{n}W_{ij}Y_{it}+\beta X_{it}+\varepsilon_{it}+\mu_{i}+\nu_{t}$$

where *Y*_*it*_ is the dependent variable, denoting the carbon emission performance of province *i* in year *t*. *X*_*it*_ is the independent variable, denoting the centrality characteristics of the carbon information disclosure network of province *i* in year *t*, and *ρ* is the spatial autoregressive parameter. *W*_*ij*_ is the element in the spatial weight matrix; *μ*_*i*_ and *ν*_*t*_ respectively denote the individual and time fixed effects, and *ε*_*it*_ is the residual perturbation term.

Spatial Durbin model (SDM): The SDM model assumes that spatial spillover effects come from the independent variables. This study shows that local carbon emission performance is influenced by the characteristics of carbon information disclosure networks in neighbouring regions. The SDM model is shown below:

(5) $$Y_{it}=\rho \sum_{j=1}^{n}W_{ij}Y_{it}+\beta X_{it}+\theta \sum_{j=1}^{n}W_{ij}X_{it}+\varepsilon_{it}+\mu_{i}+\nu_{t}$$

where *θW*_*ij*_ is the effect of neighbourhood independent variables on the dependent variables; *W*_*ij*_ is a 30 × 30 spatial weight matrix; other symbols are synonymous with the SAR model.

Spatial error model (SEM): The SEM model assumes that spatial spillovers are due to external random shocks or omitted variables that impact the explanatory variables. It is manifested in this study as the spatial dependence of carbon emission performance through a random disturbance term. The SEM model is specified as

(6) $$Y_{it}=\beta X_{it}+\pi_{it}+\mu_{i}+\nu_{t}$$

(7) $$\pi_{it}=\lambda \sum_{j=1}^{n}W_{ij}\pi_{it}+e_{it}$$

where λ is the spatial error term coefficient, reflecting the local impact of the error terms of the dependent variables in neighbouring regions; *e*_*it*_ is the residual disturbance term, and the other symbols are synonymous with the SAR model.

### 3.2 Data collection and variables measurement

The data related to industrial enterprises in 30 provincial regions in mainland China (Tibet was excluded due to the large amount of missing data) from 2012 to 2021 are selected as research samples. The relevant raw data come from the China Energy Statistical Yearbook, China Science and Technology Statistical Yearbook, China Statistical Yearbook, China Industrial Statistical Yearbook and the China Stock Market and Accounting Research (CSMAR) database. Considering the comparability of the data, the data on GDP are converted into 2012 constant price.

#### 3.2.1 Variables for social network analysis

Carbon information disclosure (CID). Drawing on existing studies [49,50], this paper calculate an evaluation index system for carbon information disclosure quality (details are shown in Table 1). In this evaluation index system, the reasons for considering ISO9001 and ISO14001 Certification when reflecting enterprises' carbon emissions regulation and certification are as follows: 1) ISO9001 Certification is one of the important factors reflecting enterprises' carbon emissions and carbon information disclosure. Enterprises can strengthen enterprise quality management, optimize costs and improve enterprise performance through the implementation of Quality Management System, which offers the economic basis for corporate environmental management, energy saving and carbon reduction. 2) ISO14001 Environmental Management System Certification encourages enterprises to adopt green technology and clean production methods to realize green development. Therefore, ISO14001 Certification is an effective manifestation of enterprises’ environmental management and carbon management. The formula for carbon information disclosure is

(8) $$CID_{i}=\frac{\sum CIDP_{i}}{MCID}$$

where *CID*_*i*_ refers to the carbon information disclosure quality of enterprise *i*. *∑CIDP*_*i*_ refers to the sum of the scores of all the disclosure items of enterprise *i*, and *MCID* refers to the sum of the highest scores of all the disclosure items. Table 1 shows that the value of *MCID* in this paper is 14. The carbon information disclosure is calculated by organising the relevant data in the CSMAR database.

**Table 1:** Carbon information disclosure assessment system.

Type	Disclosure items	Instructions
Carbon emissions regulation and certification	Environmental emergency	Having environmental pollution incident records 1point, otherwise 0 point
	ISO14001 Certification	Having been certified records 1 point, otherwise 0 point
	ISO9001 Certification	Having been certified records 1 point, otherwise 0 point
Low-carbon goals and concepts	Environmental goals	Disclosing environmental goals records 1 point, otherwise 0 point
	Environmental concepts	Disclosing environmental concepts records 1 point, otherwise 0 point
Carbon emissions management	Environmental management system	Disclosing environmental management system records 1 point, otherwise 0 point
	Environmental education and training	Disclosing environmental education and training records 1 point, otherwise 0 point
	“Three simultaneous” system	Disclosing “Three simultaneous” system records 1 point, otherwise 0 point
	Key pollution monitoring Units	Being key pollution monitoring units records 1 point, otherwise 0 point
Carbon emissions governance	Pollutant emission	Meeting pollutant emission standards records 1 point, otherwise 0 point
	Waste gas emission reduction and treatment	Quantitative descriptions records 2 point, qualitative descriptions records 1 point, no descriptions records 0 point
Carbon emission accounting	CO_2_ emissions	Quantitative descriptions records 2 point, qualitative descriptions records 1 point, no descriptions records 0 point

Notes: When calculating ISO14001 and ISO 9001 Certification, the data before 2015 take ISO14001: 2004 version and ISO9001: 2008 version as standards, and the data for 2015 and subsequent years take ISO14001: 2015 version and ISO9001: 2015 version as standards.

#### 3.2.2 Variables for spatial measurement models

(1) Dependent variables

Carbon emission performance (CEP): this paper refers to the efficiency evaluation indexes for carbon emissions of heavily polluting industries constructed by Yang and Niu [51]. This paper selects intra-mural expenditure on R&D, the number of R&D personnel and the total industrial energy consumption in each province to represent capital, labour, and energy input, respectively. The sales revenue of new products and the number of patent applications are taken as desired outputs, and carbon dioxide emissions are taken as undesired outputs. Moreover, the undesirable-SBM model calculates the carbon emission performance of industrial enterprises with green technologies. Combined with the IPCC greenhouse gas accounting method and the relevant parameters published by China, the specific formula for calculating CO_2_ emissions is as follows:

(9) $$C=\sum_{i=1}^{5}C_{i}=\sum_{i=1}^{5}E_{i}\times NCV_{i}\times CC_{i}\times COF_{i}\times \frac{44}{12}$$

Five kinds of energy sources, such as raw coal, coke, gasoline, diesel and fuel oil, are selected to calculate CO_2_ emissions. Here, *i* denotes the type of energy source, *C* denotes the total amount of carbon dioxide emission and *E*_*i*_ denotes the consumption of energy *i*. *NCV*_*i*_ is the lowest calorific value, *CC*_*i*_ is the amount of carbon per unit of calorific value and *COF*_*i*_ is the carbon oxidation factor. Combined with the actual situation in China, the standardised conversion factor, NCV, CC, and COF are taken from the China Energy Statistical Yearbook and the Guidelines for Provincial-level Greenhouse Gas Inventories. The related values are shown in Table 2.

(2) Independent variables

**Table 2:** Indicators for the calculation of carbon dioxide emissions.

Energy	Standardized conversion factor (kgce/kg)	Lowest calorific value (kJ/kg)	Amount of carbon per unit of calorific value (tc/TJ)	Carbon oxidation factor
Raw coal	0.7143	20934	27.4	0.94
Coke	0.9714	28470	29.5	0.93
Gasoline	1.4714	43124	19.8	0.98
Diesel	1.4571	42705	20.2	0.98
Fuel oil	1.4286	41868	21.1	0.98

Out-degree centrality, in-degree centrality, and betweenness centrality: The independent variables come from the standardised values in the output of social network analysis, mainly reflecting the characteristics of carbon information disclosure network centrality. The good network position (out-degree, in-degree and betweenness) of different provinces in the carbon information disclosure network has an important impact on the provinces’ access to information and adjustment of environmental economic policies. Provinces occupying the centrality position can obtain the advantage of multi-source information, therefore, this paper mainly selects out-degree centrality, in-degree centrality, and betweenness centrality as independent variables.

(3) Control variables

This study follows the ideas of existing research [[52], [53], [54], [55], [56]], and control variables include the level of scientific and technological innovation (tech), industrial structure (struc), high-quality human resources (hhr), the level of urbanization (ur), government intervention (gov) and R&D personnel input (labour), which have an important influence on carbon emission performance. To a certain extent, the level of scientific and technological innovation has a supporting role in the green and sustainable development of the regional economy. Industrial structure helps to optimize resource allocation, and industrial restructuring affects the intensity of carbon emission. Urbanisation may lead to high-energy consumption and carbon emissions, but it may also lead to technological upgrading and industrial restructuring, thus helping to reduce carbon emissions. Government intervention may promote green development but may not achieve the desired effect of improving carbon emission performance. Furthermore, the quality of human resources, R&D personnel input and other technological progress issues will likely impact carbon emission performance. This paper uses the natural logarithm of the number of patents granted, the share of industrial value-added in GDP, the ratio of high school education and above to the number of employed persons, the urbanisation rate, the ratio of general public budget expenditure to regional GDP and the natural logarithm of the full-time equivalent of R&D personnel to denote the level of scientific and technological innovation (tech), industrial structure (struc), high-quality human resources (hhr), urbanisation rate (ur), government intervention (gov) and R&D personnel input (labour), respectively.

(4) Geographic distance spatial weight matrix

The geographic distance between the capital city of province *i* and province *j* is *d*_*ij*_; then, the spatial weight is *w*_*ij*_ = 1/*d*_*ij*_. The specific form of the spatial weight matrix is as follows:

(10) $$W=\left(\begin{array}{ccc} \frac{1}{d_{11}} & \ldots & \frac{1}{d_{1n}}\\ \vdots & \ddots & \vdots \\ \frac{1}{d_{n1}} & \cdots & \frac{1}{d_{nn}} \end{array}\right)$$

Table 3 shows the definition and description of main variables.

**Table 3:** Definition and description of main variables.

Type	Name	Code	Definition
Dependent variables	Carbon emission performance	CEP	Measured by undesirable-SBM model
Independent variables	Out-drgree centrality	out-degree	Calculated by UCINET
	In-degree centrality	in-degree	Calculated by UCINET
	Betweeness centrality	betweenness	Calculated by UCINET
Control variables	Level of scientific and technological innovation	tech	The natural logarithm of the number of patents granted
	Industrial structure	struc	The share of industrial value-added in GDP
	High-quality human resources	hhr	The ratio of high school education and above to the number of employed persons
	Urban rate	ur	The urbanization rate
	Government intervention	gov	The ratio of general public budget expenditure to regional GDP
	R&D personnel input	labour	The natural logarithm of the full-time equivalent of R&D personnel

## 4 Empirical results

### 4.1 Structural analysis of carbon information disclosure spatial association networks

#### 4.1.1 Analysis of network structure diagrams

**Fig. 2:**
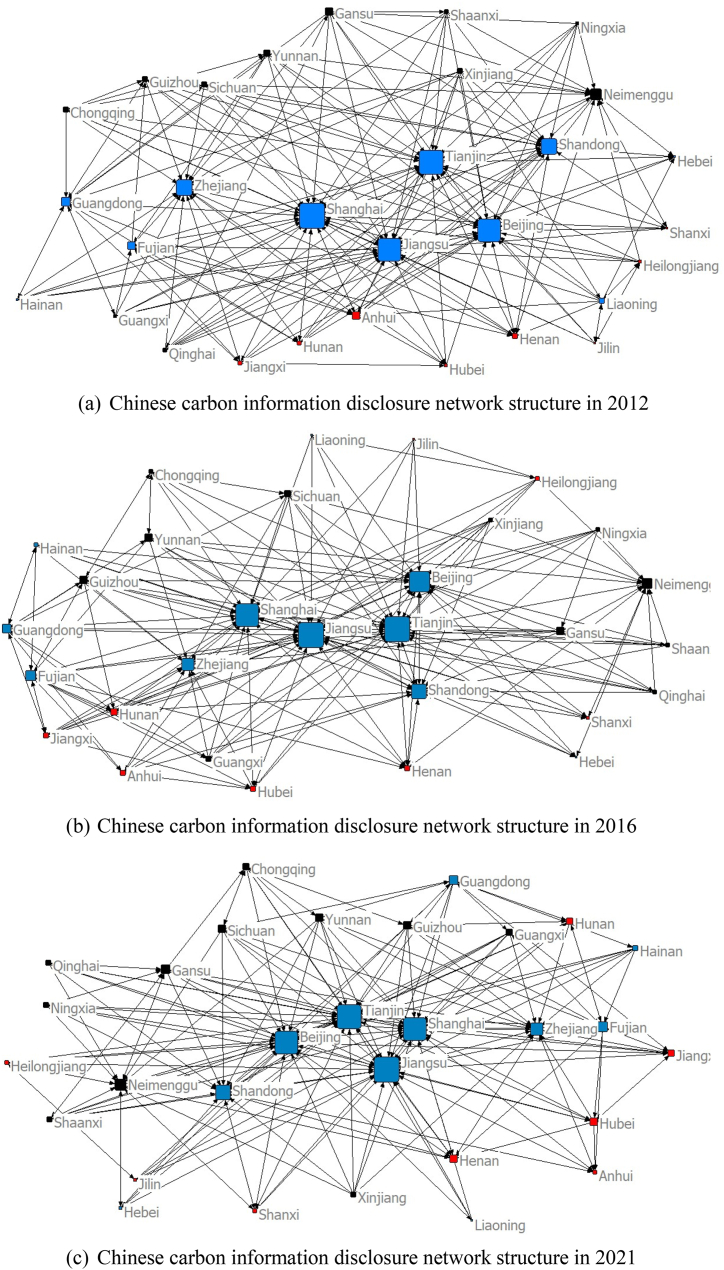
Chinese carbon information disclosure network structure in 2012, 2016 and 2021.

This paper is based on the NETDRAW function of UCINET to draw the network structure map of China's provincial carbon information disclosure, with nodes representing different provinces and the connecting lines as directed line segments representing the interaction of carbon information disclosure among provinces. Fig. 2 presents the spatial network structure of carbon information disclosure by province in 2012, 2016 and 2021. Blue nodes represent the eastern region, red nodes represent the central region and black nodes represent the western region. Fig. 2 shows that the spatial structure of China's carbon information disclosure network generally presents the characteristics of a strong connection in the eastern region and a sparse connection in the central and western regions. The eastern region is at the network's core, with a relatively strong centrality of the nodes.

#### 4.1.2 Analysis of network density

**Fig. 3:**
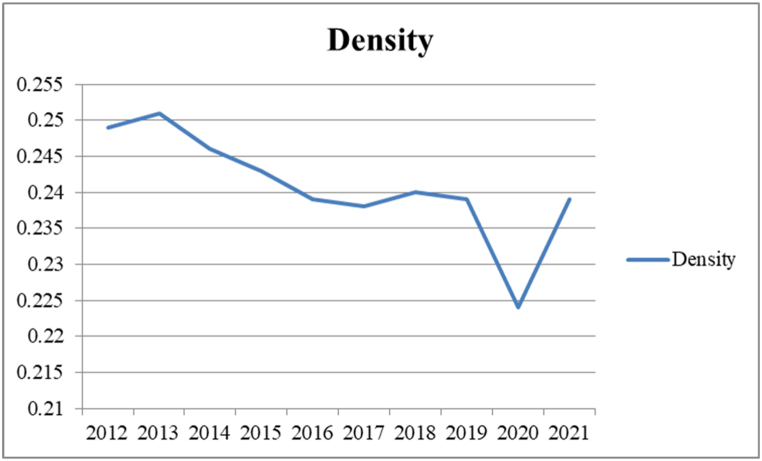
Carbon information disclosure network density in China.

Fig. 3 shows that the density of carbon information disclosure networks in China's provinces decreases and then increases from 2012 to 2021, which, to a certain extent, can illustrate the spatial and temporal evolution trend of the network structure of carbon information disclosure among regions. The overall low network density indicates that there is still much room for improvement in the carbon information disclosure of industrial enterprises in Chinese provinces.

#### 4.1.3 Analysis of degree centrality

According to Table 4, the analysis of degree centrality is as follows. Comparing the network degree centrality indicators in 2012, 2016 and 2021, it can be found that: in terms of in-degree centrality, although the top six provinces have changed, the in-degree centrality of Shanghai, Jiangsu, Tianjin, Beijing and Zhejiang have been in the top six provinces in the country. This ranking indicates that these provinces are the significant agglomeration of carbon information disclosure. Regarding out-degree centrality, Fujian and Guangdong are at the top of the list, indicating that the spillover effect of their carbon information disclosure is more pronounced, but their in-degree centrality is significantly weaker than those of the top six provinces. This ranking indicates that the clustering effect of carbon information disclosure in these provinces is weak. Combining the in-degree centrality and the out-degree centrality, the eastern provinces, such as Shanghai, Jiangsu, Tianjin, Beijing and Shandong, are located at the core of the carbon information disclosure networks and have more influence on the network, which is likely to be closely related to the geographic location and economic development level of these provinces. Overall, the distribution of the out-degree centrality is more even, while the difference in the in-degree centrality is noticeable. That is to say, the spillover effect of carbon information disclosure is more balanced than the agglomeration effect in each province.

**Table 4:** Carbon information disclosure network degree in China.

Year	2012		2016		2021	
Node	In-degree	Out-degree	In-degree	Out-degree	In-degree	Out-degree
Shanghai	25	8	23	7	23	7
Jiangsu	23	5	26	4	26	5
Tianjin	23	5	26	5	25	5
Beijing	22	5	22	5	24	6
Zhejiang	17	6	13	5	14	5
Shandong	15	7	15	7	14	7
Anhui	11	3	7	4	6	3
Neimenggu	11	7	11	5	11	6
Guangdong	10	9	7	11	6	10
Henan	9	7	8	7	9	7
Jiangxi	6	8	6	8	6	7
Fujian	4	9	5	10	6	10
Gansu	4	10	4	9	4	10
Hebei	5	5	4	5	4	5
Hubei	4	7	4	7	4	8
Hunan	4	8	4	8	4	7
Guizhou	3	8	3	9	3	10
Liaoning	3	8	1	5	1	4
Shanxi	3	6	4	6	4	6
Chongqing	3	9	3	7	4	7
Heilongjiang	2	7	2	7	1	6
Jilin	2	6	1	5	1	5
Sichuan	2	9	2	9	1	9
Yunnan	2	10	2	10	2	10
Guangxi	1	7	2	8	2	8
Hainan	1	6	1	7	1	7
Qinghai	1	8	1	7	1	7
Shaanxi	1	8	1	7	1	7
Ningxia	0	7	0	7	0	7
Xingjiang	0	9	0	7	0	7

### 4.2 Tests and results analysis of spatial econometric models

#### 4.2.1 Tests of spatial econometric models

Before spatial econometric analysis, it is necessary to test whether a spatial correlation exists between the data. Therefore, the spatial Moran's I is calculated, and the results show that the univariate Moran's I statistic of carbon emission performance is 0.044, and the corresponding P-value is 0.005. This outcome proves the reasonableness of adopting the spatial econometric models. Because the spatial autocorrelation test can only initially test the existence of spatial effects, it is necessary to further establish the spatial econometric models to analyse the spatial relationship between variables in depth. Therefore, the appropriate spatial econometric models are selected through the Lagrange multiplier (LM) test, and whether there is a fixed effect or random effect in the spatial models is tested through the Hausman test. The relevant test results are shown in Table 5.

**Table 5:** Results of LM test and Hausman test.

Test	Statistic	p-value
LM spatial error	26.451	0.000
Robust LM spatial error	80.781	0.000
LM spatial lag	0.870	0.351
Robust LM spatial lag	55.200	0.000
Hausman test	78.45	0.000

Table 5 shows that the LM and R-LM statistics of the SEM are significant at a 1 % significance level; however, the LM statistic of the spatial lag model is 0.870, which fails the significance test. Therefore, it is more reasonable to choose the SEM to test the spatial impact of the spatial association network characteristics of carbon information disclosure on carbon emission performance. Moreover, from the practical significance and econometric point of view, numerous factors affect carbon emission performance. At the same time, the independent and control variables considered in this paper are complex to cover, and all the influencing factors, and other factors not considered are included in the random perturbation term. According to the results of the Hausman test, chi2(10) = 78.45 and Prob > chi2 = 0.000, the original hypothesis is rejected to choose fixed effects.

Through the above analysis, it can be determined that an SEM with fixed effects can be chosen to analyse the impact of carbon information disclosure spatial network characteristics on carbon emission performance, and the next step is to select the type of fixed effects. This paper uses the likelihood ratio (LR) test to select the appropriate fixed effects, and the LR test hypothesises that the double fixed effects will degenerate into time fixed effects or spatial fixed effects. Table 6 shows that the LR tests all pass the 1 % significance test and reject the original hypothesis; therefore, it is more reasonable to choose the SEM ((6), (7)) under double fixed effects in this paper.

**Table 6:** Results of LR test.

	χ^2^	P-value
both VS ind	45.40	0.0000
both VS time	240.80	0.0000

#### 4.2.2 Results analysis of spatial econometric models

This study uses an SEM with double fixed effects to empirically examine the impact of spatial network characteristics of carbon information disclosure on carbon emission performance in 30 provincial-level regions in China. The results are shown in Table 7.

(1) The spatial coefficient λ of the spatial error term is negative. It passes the 1 % significance level test, indicating that a significant spatial dependence on provincial carbon emission performance. That is to say, the carbon emission performance of industrial enterprises in a province is related to the relevant factors in this province and affected by the carbon emission performance of the neighbouring regions because of the random perturbation term. Under the research conditions of this paper, this effect is negative. This phenomenon may be due to a time gap between regions implementing the environmental protection system. The strength of the construction of the system is not the same, which leads to some enterprises being constrained by the pressure of capital and supervision, and choosing to remove from the region with high environmental protection requirements to the lower environmental protection requirements below the development of the region, which will have a positive impact on the carbon emission performance of this region and negatively influence the carbon emission performance in the neighbouring regions.
(2) The spatial network characteristics of carbon information disclosure affect carbon emission performance. The parameter estimation results for the independent variables show that the out-degree centrality and betweenness centrality respectively pass the parameter significance test at the 10 % and 5 % levels, and the parameter estimates are negative. In-degree centrality does not pass the significance test, but the parameter estimates are also negative. This result indicates that, at the provincial level, the spatial network centrality characteristics of industrial enterprises' carbon information disclosure hinder improving enterprises' carbon emission performance. Meanwhile, for every 1 % increase in the network out-degree centrality of carbon information disclosure, the carbon emission performance of the region will be reduced by 0.688 %; for every 1 % increase in the network betweenness centrality degree of carbon information disclosure, the carbon emission performance of the region will be reduced by 0.012 %. For a specific province, higher carbon information disclosure network out-degree centrality and betweenness centrality imply that the region has a stronger external spillover ability in carbon information disclosure and is vital output node. On the one hand, this indicates to some extent that the government has imposed environmental regulations on enterprises, but the intensity of environmental regulations has not reached the ‘regressive effect’, and the ‘follow the cost’ effect still dominates. The enterprises have increased their investment in environmental protection, and the ‘follow the cost’ effect leads to the negative impact of carbon emission disclosure on carbon emission performance. On the other hand, occupying a central position in the carbon information disclosure network means that the province has more control over carbon emission information resources. Suppose these enterprises have the pandering behaviour of carbon information disclosure. In that case, that is to say, the disclosure of carbon emission information is not in line with the actual carbon-reduction actions; it will lead to false disclosure of carbon emission information. Reducing useful carbon emission information interferes with formulating government policies and investors' investment decisions and is not conducive to improving carbon emission performance. Although the coefficient of carbon information disclosure network in-degree centrality on carbon emission performance does not pass the significance test, it is only statistically insignificant, which does not mean it has no effect. If the confidence level is lowered, it can still be assumed that the carbon information disclosure network in-degree centrality will hinder carbon emission performance. The higher the carbon information disclosure network's in-degree centrality is, the more the information of carbon information disclosure in the network is concentrated in the province, which will have a particular impact on the change in carbon emission performance.
(3) The test results of the control variables show that the level of science and technology innovation (tech) and industrial structure (struc) passed the 1 % significance level test and the 5 % significance level test for high-quality human resources (hhr) and government intervention (gov). Meanwhile, all the coefficients are positive, which indicates that, at the provincial level, the improvement of science and technology innovation, industrial structure, high-quality human resources, and government intervention all significantly promote the carbon emission performance of industrial enterprises in the province. In addition, it is worth noting that the effect of urbanisation rate on carbon emission performance in this paper is positive but not significant. The possible explanation is that China's existing urbanisation model is primarily an unsustainable traditional industrial model. In the current era of green economic development, the required urbanisation is a new type of urbanisation that undergoes a green transformation, which may take much longer before it produces results; thus, it is difficult to have a significant impact on the improvement of carbon emission performance in the short term. In addition, the results of this paper show that R&D personnel input has a significant negative impact on carbon emission performance, which may be due to the mismatch between R&D input and R&D human capital input. When this mismatch occurs, it will be unfavourable to the development of technological progress, which is not conducive to improving carbon emission performance. Liang [57] points out that the mismatch between R&D funding and R&D human capital investment will lead to R&D investment's ‘Solow paradox’, which is not conducive to improving industrial green total factor productivity.
(4) Robustness test. The network centrality indicator of the independent variables is replaced with the raw carbon information disclosure data to verify the reliability of the empirical results. The relevant results are shown in Table 8, and the robustness test results are consistent with the previous regression results, proving the robustness of the research findings.

**Table 7:** Estimation results of spatial error models.

Independent variables	Coefficients	Z-value	Control variables	Coefficients	Z-value	λ	Sigma^2^
out-degree	−0.688^a^	−1.70	tech	0.194^c^	4.49	−0.963^c^	0.0168^c^
in-degree	−0.421	−1.16	struc	0.991^c^	2.82		
betweenness	−0.012^b^	−2.21	hhr	1.091^b^	2.52		
			ur	0.588	0.99		
			gov	1.059^b^	2.42		
			labour	−0.209^c^	−4.82		

a *p* < 0.1.

b *p* < 0.05.

c *p* < 0.01.

**Table 8:** Results of the robustness test.

Independent variables	Coefficients	Z-value	Control variables	Coefficients	Z-value	λ	Sigma^2^
CID	−0.567^a^	−1.95	tech	0.185^c^	4.29	−1.012^c^	0.0172^c^
			struc	0.774^b^	2.22		
			hhr	1.057^b^	2.37		
			ur	0.427	0.72		
			gov	1.639^c^	4.02		
			labour	−0.145^c^	−3.27		

a *p* < 0.1.

b *p* < 0.05.

c *p* < 0.01.

## 5 Discussion

### 5.1 Comparison with existing studies on carbon information disclosure and carbon emission performance

This study analyses the impact of spatial association network of carbon information disclosure on carbon emission performance from the perspective of spatial association. Through empirical analysis, the following important conclusions are drawn.

(1) Spatial dependence of carbon emission performance exists among different provinces. This finding indicates that a province's carbon emission performance affected by the relevant factors in the province, which further validates the existing research that carbon productivity exhibits significant spatial dependence [58]. This finding has important implications for the government promoting carbon emission performance. It requires the government to fully consider the province's economic interests, environmental benefits and the impact of policies in neighbouring regions when formulating policies.
(2) A negative relationship exists between the network's in-degree centrality, out-degree centrality and betweenness centrality of carbon information disclosure network and carbon emission performance. This results indicates that carbon information disclosure negatively affects carbon emission performance, which is consistent with the previous findings of Hao et al. [33]. Hao et al. found that the current environmental control measures do not achieve the expected goal of controlling and reducing pollution.

The above findings are of great significance to the government in balancing economic development and environmental protection. First, at the practical level, it provides the guidance needed to improve carbon emission performance. For example, the government can understand the key provinces of the current carbon information disclosure and further adjust the relevant policies through the implementation effect of the current carbon information disclosure policy, thus laying the foundation for promoting carbon information disclosure policy on carbon emission performance. Second, from a theoretical perspective, this finding further expands the theory of the relationship between environmental regulation and economic development from the perspective of social network theory.

Nevertheless, there are some differences in findings compared to previous studies. Although one of the carbon information disclosure indicators (in-degree centrality) selected in this paper negatively affects carbon emission performance, this effect is insignificant. This result suggests that the effect of information reception of network nodes in the carbon information disclosure process on carbon emission performance may not be significant, which may be due to sample selection, methodology or other factors that are not considered. For example, only industrial enterprises in various provinces are selected as samples, which may lead to the results not being generalisable in other fields. To better explain the differences in the results, future research could consider expanding the sample size and adopting different methodologies for further validation.

### 5.2 Limitations and future prospects

This paper empirically analyses the conclusion that the structural characteristics of carbon information disclosure networks can affect carbon emission performance. However, the limitations of this study require attention. First, due to data limitations, this inquiry cannot understand the reasons for information flows in carbon information disclosure networks. Second, although the impact of spatial correlation networks of carbon information disclosure on carbon emission performance is discussed, the reality is that the advancement of carbon information disclosure policies varies from province to province, and lax regulation can create incentives for firms to make false disclosures, which may have a substantial impact on carbon emission performance. Third, this paper only examines carbon information disclosure and carbon emission performance in China and does not explore the relevant situation in other countries.

Considering the above limitations, further research could be explored in the following aspects. First, future research could examine the impact of carbon information flow on carbon emission performance for different reasons. For example, data can be collected from representative enterprises in each province through large-scale questionnaires or field research to conduct further research. Second, future research can further improve the evaluation index system of carbon information disclosure quality to eliminate the impact of false disclosure on carbon emission performance and more comprehensively analyse the relationship between carbon information disclosure and carbon emission performance. Third, future research could construct carbon information disclosure networks among different cities in other countries and analyse their impact on carbon emission performance. One important future direction of this study is analysing the linkages of carbon information disclosure among different countries from an international perspective to promote global carbon emission reduction.

## 6 Conclusions and recommendations

### 6.1 Conclusions

We use the data related to the disclosure of corporate social responsibility reports in the CSMAR database to calculate the quality of carbon information disclosure of A-share listed industrial enterprises in provinces. This paper constructs a social network analysis model to analyse the spatial network characteristics of carbon information disclosure in China's provincial areas and adopt an SEM to analyse the spatial dependence of carbon emission performance and the impact of the centrality characteristics of the carbon information disclosure network on the performance of carbon emission. We obtain the following conclusions.

First, the network density of carbon information disclosure in China decreases and then increases. The overall network density is low, indicating substantial room for improvement in the communication and exchange of carbon information disclosure in China's provincial areas. Second, the carbon emission performance between different provinces will have spatial dependence due to the existence of random perturbation terms. Finally, spatial network characteristics of carbon information disclosure can negatively affect carbon emission performance, with network out-degree centrality and betweenness centrality being more significant than network in-degree centrality. This paper's results can help the Chinese government understand the implementation effect of the current carbon information disclosure policy. At the same time, this paper provides a theoretical foundation and decision-making basis for utilising carbon information disclosure to improve carbon emission performance from the perspectives of regional carbon information flow and the flow of economic factors.

### 6.2 Recommendations

This paper makes the following possible policy recommendations in response to the above research.

(1) It is necessary to deepen the reform of the carbon information disclosure system and improve the quality of carbon information disclosure. This study shows that the carbon information disclosure at the current stage does not effectively promote the improvement of carbon emission performance. The reason for this phenomenon may be that the lack of unified and reasonable carbon information accounting standards leads to the low quality of corporate carbon information disclosure. Therefore, the government should specify carbon information disclosure content and promote the standardised application of carbon information among regions. For example, further standardising the format and accounting methods of corporate carbon information disclosure could guarantee the authenticity and comparability of carbon information. The government should implement a mandatory carbon information disclosure system in phases, gradually expanding it from provinces at the centre of the carbon information disclosure network to peripheral provinces. This measure can utilise the control and information transmission capabilities of the central provinces. In addition, the government should regulate strictly to avoid speculative behaviours such as false disclosure and realize the flow of high-quality carbon information throughout the country.
(2) Strengthening cross-regional cooperation on environmental and economic policies is conducive to avoiding the negative impacts of policies in neighbouring regions and achieving mutual benefits. The carbon emission performance of industrial enterprises in a province is negatively affected by the carbon emission performance of the neighbouring regions. This phenomenon may be due to a time gap between regions implementing the environmental protection system. Therefore, strengthening the linkage of carbon information disclosure between peripheral and core regions and exploring the path of multi-centre synergistic development are all conducive to improving carbon information interaction. This approach could alleviate the negative impact of time differences in environmental policy implementation in different regions on carbon emission performance. Moreover, cooperative environmental governance in neighbouring regions can reduce competition for economic resources. For example, neighbouring regions could exchange low carbon technologies and talents and use Internet for establishing a carbon information disclosure database to make the data open and transparent.
(3) To promote the carbon information disclosure policy in the direction of promoting economic development, the government should improve the design of the carbon information disclosure system. First, the government could revise the environmental policies and eliminate the barriers to the flow of information and economic factors between regions at the institutional level. Second, the government could establish a reward and punishment mechanism for carbon information disclosure. Such a mechanism could provide a sound policy support to reward positive carbon information disclosure behaviours and to punish false disclosure behaviours. Third, establish a coordinated regulatory mechanism for corporate carbon information. The public could supervise the carbon reduction actions of enterprises, the media could expose enterprises with poor carbon information disclosure, and environmental enforcement departments could reduce the frequency of law-enforcement for enterprises with good carbon information disclosure.

## Ethical approval

Not applicable.

## Data availability statement

The data associated with this study has not been deposited into a publicly available repository, because the authors do not have permission to share data. However, the data that support the findings of this study are available from the China Energy Statistical Yearbook (https://www.stats.gov.cn/), China Science and Technology Statistical Yearbook (https://data.cnki.net/), China Statistical Yearbook (http://www.stats.gov.cn/sj/ndsj/), China Industrial Statistical Yearbook (https://data.cnki.net/)and the CSMAR database (http://www.gtarsc.com/).

## Funding

This research was funded by the Project of Liaoning Provincial Federation Social Science Circles of China (no. L20BGL047).

## CRediT authorship contribution statement

**Tianjiao Jiang:** Writing – review & editing, Writing – original draft, Visualization, Validation, Software, Methodology, Investigation, Formal analysis, Data curation, Conceptualization. **Hua Li:** Supervision, Project administration, Funding acquisition. **Qiubai Sun:** Supervision, Project administration, Funding acquisition.

## Declaration of competing interest

The authors declare that they have no known competing financial interests or personal relationships that could have appeared to influence the work reported in this paper.

## Figures and Tables

**Table:** List of Abbreviations

CSMAR	the China Stock Market and Accounting Research
DEA	Data Envelopment Analysis
GDP	Gross Domestic Product
LM	Lagrange Multiplier
LR	Likelihood Ratio
R&D	Research and Development
SAR	Spatial Autoregressive Model
SBM	Slacks-Based Measure
SDM	Spatial Durbin Model
SEM	Spatial Error Model
SFA	Stochastic Frontier Approach
